# A new variant of Choledochal cyst-type VI: a rare case presentation and review of the literature

**DOI:** 10.1093/jscr/rjad545

**Published:** 2023-12-19

**Authors:** Yufan Bai, Chunmei Li, Jihong Tang, Min He

**Affiliations:** Department of Hepato-Biliary-Pancreatic Surgery, The Second Affiliated Hospital of Kunming Medical University, Kunming, China; Department of Hepato-Biliary-Pancreatic Surgery, The Second Affiliated Hospital of Kunming Medical University, Kunming, China; Department of Hepato-Biliary-Pancreatic Surgery, The Second Affiliated Hospital of Kunming Medical University, Kunming, China; Department of Hepato-Biliary-Pancreatic Surgery, The Second Affiliated Hospital of Kunming Medical University, Kunming, China

**Keywords:** choledochal cysts, cystic duct cysts, the multidisciplinary team

## Abstract

A 53-year-old male patient with a previous diagnosis of dilatation of the common bile duct was admitted to the hospital due to recurrent episodes of vague epigastric pain over a 4-month period. After undergoing abdominal CT, MRI, MRCP, ERCP examinations, together with joint diagnosis by the radiology department and the gastroscopy unit, the diagnosis of a cystic dilatation of the common bile duct was excluded, and to preliminarily diagnose as cystic lesion at the hepatoduodenum ligament. A nasobiliary tube was preset before the surgery, and it was found that the gallbladder, the cyst, and the common bile duct were connected in sequence during the surgery, leading to the definitive diagnosis of biliary cyst of the cystic duct. During the surgery, the anatomical position of the common bile duct was accurately identified, avoiding iatrogenic biliary injury and preserving the integrity of the common bile duct structure. The patient recovered and was discharged from the hospital on the 14th postoperative day. Cystic duct cysts are a relatively new and rare condition. This case demonstrates that clinical decision-making by a multidisciplinary team is of great significance for such diseases, and preoperative assessment of the anatomical relationship between cystic dilation lesions in the hepatic portal region and the biliary system and gallbladder is also crucial.

## Introduction

Choledochal cysts refers to the dilatation of the bile duct that involve the extrahepatic or intrahepatic bile duct [1]. Currently, Todani classification is a widely accepted classification for Choledochal cysts, which includes five types [2]. However, these five types do not seem to include biliary cysts of the cystic duct well [3]. Biliary cysts of the cystic duct are very rare [2]. Bode and Serena had identified this disease as a new type of Choledochal cysts in 1983 and 1991, which named cystic duct dilatation cysts, and classified as Todani type VI [4]. More and more scholars have also accepted that cystic duct dilatation cysts are included in Type VI Choledochal cysts [5–7]. This article reports a case that was previously misdiagnosed as dilatation of the common bile duct, but eventually diagnosed as Cystic duct cysts, in order to provide reference for the clinical diagnosis and treatment of cystic duct cysts.

## Case report

**Figure 1:**
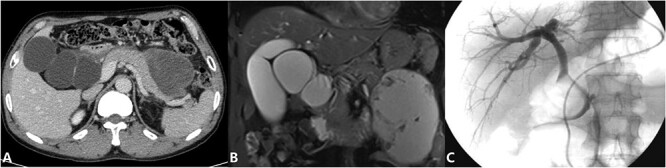
(Preoperative image) A: CT; B: MRCP; C: ERCP.

The patient is a 53-year-old man who was admitted to the hospital due to recurrent episodes of vague epigastric pain over a 4-month period. The patient had been diagnosed with Choledochal cysts and pancreatic pseudocyst in a local hospital before coming to seek treatment in our hepatological surgery department. Clinical evaluation revealed no yellowing of the skin or sclera, no abdominal wall varices, a soft abdomen without tenderness, and no abnormal masses felt. The liver and spleen were not palpable. The routine laboratory tests also were unremarkable. CT scan (Fig. 1A) showed cystic dilation lesion of the extrahepatic bile duct, which the largest one ~6.3 × 5.6 cm, and a mass of mixed density shadow in the pancreatic body and tail that measuring ~10.3 × 7.8 cm. MRI and MRCP (Fig. 1B) revealed multiple cystic dilation lesions in the common bile duct, which was considered as Choledochal cyst type I and a pseudocyst forming in the body and tail of the pancreas. ERCP (Fig. 1C) revealed that there is not dilation of the bile ducts and the cystic duct was partially developed, but the gallbladder was not developed. A multidisciplinary consultation was conducted by the hepatological surgery department in collaboration with the radiology department and endoscopy unit. The diagnosis is suspected to be a cystic lesion at the hepatoduodenal ligament, and it has been decided to perform preoperative ERCP followed by nasobiliary tube placement.

**Figure 2:**
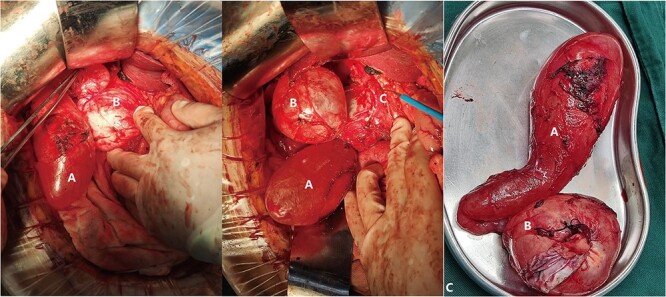
(Intraoperative image) A: Gallbladder; B: Cystic Duct Cyst; C: Common Bile Duct.

After the patient signed an informed consent, an exploratory laparotomy was performed. During the surgery, because nasobiliary tube was placed in the common bile duct before the operation, the anatomical position of the common bile duct was clearly exposed, it was found that the cyst was closely related to the common bile duct. In order to avoid damage to the common bile duct and other tissues, we proceeded to dissect the gallbladder retrograde from the fundus of the gallbladder. After the gallbladder was separated from the gallbladder bed, we found that the dilated bile duct neck was connected to the cystic lesions. According to the position of the nasobiliary tube, the anatomical relationship among the gallbladder, the cystic lesions, and the common bile duct could be clarified, and the cyst was carefully separated from the common bile duct. The gallbladder, the gallbladder neck and the cystic lesions were found to be connected in turn, and they finally merged with the common bile duct. During the surgery, the contents of the gallbladder and the cystic lesions were aspirated with a syringe, which were clear and transparent fluids. When the cyst was removed, an opening about 1 cm in length was found at the junction of the common bile duct and the cystic lesions, and bile flowed out from the opening. The opening was sutured primarily. The abdominal cavity was explored again to determine the location of the pancreatic pseudocyst, and pancreatic pseudocyst jejunostomy anastomosis was performed. During the operation, cystic duct dilatation cysts and pancreatic pseudocyst were diagnosed. The intraoperative findings are shown in Fig. 2.

**Figure 3:**
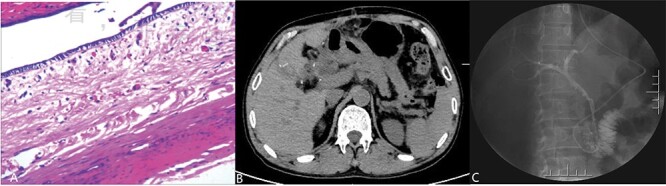
(Postoperative image) A: Pathological examination revealed a cyst with a fibrous wall, dense chronic inflammatory infiltration; B: CT after operation; C: Radiography through nose-bile tube after operation.

On the 14th day after the operation, the patient did not experience any recent postoperative complications, and the contrast agent successfully entered the intestinal cavity through the common bile duct during fluoroscopy (Fig. 3). And the nasobiliary duct was removed. The patient recovered and was discharged.

## Discussion

Choledochal cysts is a rare type of biliary malformation, and cystic duct cysts that do not involve other biliary systems are even rarer [8, 9]. Clinical symptoms of cystic duct cysts are usually similar to other types of Choledochal cysts. According to anatomical position and size of the cystic lesions, clinical symptoms range from asymptomatic to abdominal pain, abdominal mass, and jaundice [4, 10]. However, clinical symptoms lack specificity, so preoperative identification is not highly accurate and often falsely reported as other types of Choledochal cysts in imaging examinations. Less than half of the patients can be correctly diagnosed before surgery [9]. In most cases, the condition needs to be confirmed by intraoperative diagnosis [10–13]. With the development of medical technology and improvement of diagnosis and treatment mode, an increasing number of patients can obtain a correct diagnosis through MRI, MRCP, or ERCP to clarify the structure of the biliary system [1, 2, 10, 14]. Cystic duct cysts are significantly different from gallbladder malformations such as double gallbladder in structure, and there is no independent duct connecting with the common bile duct [13]. The key to the diagnosis of Cystic duct cysts is to clarify the structure of the biliary system, especially paying attention to cystic lesions structures with dilation and no blood supply near the hepatic portal region [5]. In this case, the postoperative pathological examination reported that the contents of the gallbladder and cystic duct cyst were clear liquid, the gallbladder wall had chronic inflammation, and the fibrous tissue of the cyst wall was hyperplasia, but the cystic duct cyst was not communicated with the common bile duct. It was considered that after the formation of the cyst wall, it led to the closure of the junction between the cystic duct and the common bile duct with the enlargement and compression of the cystic lesions and the existence of inflammation, which led to poor drainage of the gallbladder and the cystic lesions and persistent inflammation. At the same time, it also increased the difficulty of preoperative diagnosis.

At present, the etiology of cystic duct cysts is still unclear. There are usually two theories. One is that cystic duct wall develops unevenly due to the lack of ganglion cells or the abnormal development of embryonic cells during embryonic development. Second, abnormal dilatation of cystic duct caused by abnormalities at the junction of the pancreaticobiliary duct or reflux of pancreatic juice [4, 8, 15]. The cancer risk in bile duct cysts would be increasing with age [16]. Therefore, we recommend surgical resection after confirming the diagnosis of bile duct cysts, and the surgical approach should be selected according to different case. Michaelides et al. had described some cases with the dilatation of the common hepatic duct, common bile duct and dilatation of the central portion of the cystic duct, and they have proposed the classification of this type of Choledochal cyst as Todani type ID [17]. There is also reports that dilatation of both cystic duct and common bile duct without the involvement of common hepatic duct was categorized as Todani type VI B, and which had isolated cystic dilatation was classified as Todani type 6A.That classification is significant for the management of type 6A is cholecystectomy and type 6B is complete excision of common bile duct with hepaticojejunostomy [18]. The surgical approach should vary in depending on the different classifications and should be selected based on the location, size, and intraoperative adhesions of the cysts. And our case is simple cholecystectomy. Furthermore, multidisciplinary teams have attracted more and more attention in clinical diagnosis and treatment, clinical decision-making by a multidisciplinary team is of great significance for surgical plans and perioperative management plans, which can provide better treatment outcomes for patients [3]. In this case, specialists of the departments of hepatobiliary surgery, radiology, and endoscopy deeply analyzed and reviewed the preoperative clinical features from their respective professional perspectives, and proposed doubts, and scrutinized details, ultimately forming a scientific and complete preoperative clinical diagnosis to avoid clinical misdiagnosis, and the multidisciplinary team decision avoided iatrogenic common bile duct injury and played an active role in postoperative bile duct decompression and preventing bile leakage and common bile duct stricture that to that the preoperative placement of nasobiliary duct in the common bile duct provides a reference for identifying the anatomical relationship between the cyst, the common bile duct, the gallbladder, and surrounding tissues and organs during the operation. It had significant benefits for the patient prognosis and treatment.

## Conclusions

Cystic duct cysts is a relatively rare disease of the biliary system. It cannot be diagnosed simply on the basis of symptoms and signs, and requires sufficient imaging and laboratory tests. As demonstrated in this case, differential diagnosis of cystic duct cysts is crucial to the selection of surgical approach and to the prognosis of the patient. Therefore, for cystic dilation lesions without blood supply in the hepatic portal region, it is necessary to assess their anatomical relationship with the biliary tract and gallbladder as much as possible, and to attach importance of decisions made by the multidisciplinary team, to strengthen communication among different disciplines before surgery. It improves scientificity and effectiveness of clinical diagnosis and treatment, avoids or reduces misdiagnosis and surgical complications or accidents.
